# The Influence of Biostimulants Used in Sustainable Agriculture for Antifungal Protection on the Chemical Composition of Winter Wheat Grain

**DOI:** 10.3390/ijerph192012998

**Published:** 2022-10-11

**Authors:** Ewa Szpunar-Krok, Joanna Depciuch, Barbara Drygaś, Marta Jańczak-Pieniążek, Katarzyna Mazurek, Renata Pawlak

**Affiliations:** 1 Department of Crop Production, University of Rzeszow, Zelwerowicza 4 St., 35-601 Rzeszow, Poland; mjanczak@ur.edu.pl; 2 Institute of Nuclear Physics, Polish Academy of Sciences, 31-342 Krakow, Poland; joanna.depciuch@ifj.edu.pl; 3 Department of Bioenergetics, Food Analysis and Microbiology, Institute of Food Technology and Nutrition, College of Natural Science, University of Rzeszow, Ćwiklińskiej 2D St., 35-601 Rzeszow, Poland; badrygas@ur.edu.pl; 4 PTWP SA Warsaw Branch, Jana Pawła II 29 St., 00-867 Warsaw, Poland; katarzyna.mazurek@ptwp.pl; 5 Biostyma Sp. z o.o., Sikorskiego 38 St., 62-300 Września, Poland; renata.pawlak@biostyma.pl

**Keywords:** plant protection, biostimulants, *Triticum aestivum* L., grain quality, Raman spectroscopy

## Abstract

Field studies were conducted from 2016 to 2019 (south-eastern Poland; 49°58′40.6″ N 22°33′11.3″ E) with the aim to identify the chemical composition of winter wheat grain upon foliar application of biostimulants, of which PlanTonic BIO (containing nettle and willow extracts) showed antifungal activity. The main chemical compositions and their spatial distribution in wheat grain were characterized by Raman spectroscopy technique. It was established that applied biostimulants and hydro-thermal conditions changed the chemical composition of the grain during all the studied years. A similar chemical composition of the grain was achieved in plants treated with synthetic preparations, including both intensive and extensive variants. The second group, in terms of an increase in fatty acid content, consists of grains of plants treated with biostimulants PlanTonic BIO, PlanTonic BIO + Natural Crop and PlanTonic BIO + Biofol Plex. The future of using biostimulants in crop production, including those containing salicylic acid and nettle extracts, appears to be a promising alternative to synthetic crop protection products.

## 1. Introduction

Wheat is among the most significant crops grown worldwide, with a cultivated area of 219 million hectares [1,2]. The plant is important because of its high yield, chemical composition and technological properties of the grain [3,4]. Wheat grain is rich in carbohydrates and has a higher protein content than other grains, and contains large amounts of minerals (Zn, Fe) and vitamins, making it a valuable food source [5,6]. Wheat flour has unique viscoelastic properties that allow it to be processed as dough for bread, pasta and other food products due to the presence of the gluten fraction. Gluten is a mixture of proteins formed after dough hydration and is responsible for its structure [7,8]. The main goal of growing wheat is not only to obtain a high grain yield but also to improve its quality. Grain yield and quality depend on the interaction between environmental, agronomic and biological factors [9,10]. Weather conditions and, in particular, the distribution of precipitation and temperatures prevailing during the growing season largely contribute to the formation of grain yield and quality [11,12]. The growth and development of plants grown under stressful conditions, such as drought, waterlogging, excessively high or low temperatures, and deficiency or excess mineral compounds in the substrate that negatively affect their metabolism, leading to weakened growth and, consequently, reduced yields [13]. Most grains show strong sensitivity to abiotic stress early in pollen development [13,14]. This can result in irregularities at the stage of grain formation and unfavourable changes in the chemical composition of the grain. These parameters are particularly adversely affected by fungal diseases and pests, the presence of which is a major constraint on wheat production [15].

With the growing interest in reducing the use of chemicals in plant cultivation, such as fertilizers and pesticides, a recent priority has been to find environmentally friendly ways to promote plant growth and development and increase crop yields. The increase in consumer demand for foods that do not contain potentially toxic residues is also not insignificant [16]. Therefore, the use of natural plant growth enhancers [17], referred to as biostimulants, which can be made from a variety of products (seaweeds, protein hydrolysates, humic substances, and microorganisms [18,19,20]), and which improve crops without causing unwanted side effects, is becoming increasingly important [21,22]. Biostimulants can perform numerous agronomic functions. Their use in crop cultivation contributes to increased crop yields while reducing dependence on chemical fertilizers. They influence basic processes and defence mechanisms in plants, enabling them to maintain homeostasis to ensure long-term adaptation, medium-term acclimatization and short-term response to changing environmental conditions [22]. This makes them safer for the environment and contributes to sustainable crop production [23]. They differ from fertilizers in that they do not directly provide nutrients but promote in plants an improvement in the efficiency of nutrient uptake and assimilation [24], grain yield quality traits [25], and increase tolerance to biotic stresses (pest and pathogen activity) and abiotic stresses (drought, frost, salinity, heavy metal environmental pollution, etc.) [18,21,26,27,28]. Metabolites in biostimulants protect plants from environmental stresses, mainly by activating plant secondary metabolite pathways and mobilizing signalling molecules to activate defence responses [29]. The role of biostimulants in crop cultivation is growing, as indicated by the value of the global market for these products [30].

Biostimulants showing antifungal activity include salicylic acid (SA) and plant extracts, including aqueous extracts of nettle (*Urtica dioica* L.). SA is a phenolic compound synthesized by plants. In the conducted experiment, SA and extract of nettle were included in PlanTonic BIO.

Knowledge of the chemical composition of cereal grains, including wheat protected with biostimulants showing antifungal activity, analyzed using Raman spectroscopy, is still insufficient so far, so the present study was undertaken. Raman spectroscopy is a technique used in biological, biomedical, food and agricultural research that allows a simultaneous analysis of various chemical compounds and an evaluation of molecular changes occurring in the objects under study [31,32]. It involves measuring the radiation of so-called Raman scattering (inelastic scattering of photons); it is a method for studying the rotational and oscillatory spectra of molecules. With Raman scattering, it is possible to obtain information on the structure of a molecule, which allows the chemical and structural characterization and identification of complex biological material. The main advantages of this analytical technique include the accuracy of the measurements, the large amount of information obtained (at a relatively low cost), the ability to examine the sample without complex preparation and processing, and non-destructiveness [33,34].

Previous field studies with biostimulants are based mainly on the effect of these products on crop yields [35]; however, there is a lack of data on their effect on the chemical composition. The purpose of this research was to determine the effect of foliar application of biostimulants, including PlanTonic BIO, which contains salicylic acid and aqueous nettle extracts with antifungal activity, on the chemical composition of winter wheat grain using Raman spectroscopic technique. A comparison of the chemical composition of plant grains treated with biostimulants and plant grains treated with synthetic fungicides was also made.

## 2. Materials and Methods

### 2.1. Plant Material and Growth Conditions

The experiment with winter wheat (*Triticum aestivum* L. subsp. *aestivum*) of the cultivar Hondia (breeder DANKO Plant Breeding, Choryń, Poland; a company belonging to the National Agricultural Support Center) was conducted in the growing seasons from 2016/2017 to 2018/2019, in the village of Pełnatycze (south-eastern Poland; 49°58′40.6″ N 22°33′11.3″ E).

A factor of the experiment was the various options of plant protection against diseases caused by fungi, including protection with synthetic preparations and biostimulants. The experimental sites are summarized in Table 1, and the chemical composition of the biostimulants is given in Table 2. The biostimulant showing antifungal activity was PlanTonic BIO. Due to the phytotoxicity of SA observed on plants [36,37], plant growth stimulants were additionally used to alleviate possible plant stress after its application in selected variants (5 and 6): BioFol Plex and Natural Crop SL.

The experiment was implemented as a field experiment. Each variant of the experiment included a wheat canopy of 200 m^2^, which was divided into 4 smaller experimental units—plots (repetitions for a given variant), each of 50 m^2^.

Fertilisers were soil- and foliar-applied. The doses and dates of fertilizer application are presented in Table 3. The following amounts of nutrients were provided with the fertilizers:

- Soil application (kgha^−^^1^ year^−^^1^)—189 N, 70 P_2_O_5_, 105 K_2_O, 135 SO_3_;
- Foliar application (gha^−^^1^ year^−^^1^)—59 N, 260 P_2_O_5_, 338.5 K_2_O, 669.5 MgO, 1450.4 SO_3_, 23.65 Fe, 2.95 B, 19.55 Cu, 88.2 Mn, 9.25 Zn, 0.79 Mo.

The forecrop for winter wheat was winter rapeseed. Wheat was sown in the last weeks of September 2016, 2017 and 2018 (the optimal date for this region of Poland), at a density of 350 grains m^−2^. The seed was treated with a seed treatment (Scenic 080 FS: fluoxastrobin 37.5 gdm^−3^, prothioconazole 37.5 gdm^−3^, and tebuconazole 5.0 gdm^−3^). The crop was harvested in the second or third week of August at full maturity (BBCH 97, plant dead and collapsing).

### 2.2. Soil Conditions

According to the FAO/WRB classification [38], the soil type was Haplic Cambisol (Eutric) formed from loess. At the start of the experiment, the abundance of available forms of P (5.84–6.32 mg 100 g^−1^) and K (13.1–18.7 mg 100 g^−1^) were average, and Mg (14.5–17.1 mg 100 g^−1^) was very high.

### 2.3. Weather Conditions

Weather conditions were given according to the records of the Experimental Station for Variety Testing in Skołoszów (49°53′ N, 22°44′ E, altitude 230 m), Poland. The course of weather conditions in the research years was variable. Annual precipitation was higher in 2016 and 2019 (by 21.3 and 8.9%, respectively) and lower in 2017 (by 6.3%) compared to the average for 1980–2015, while in 2018, annual precipitation was close to the multi-year average. In all years of the study, the average annual air temperature was higher than average for the years 1980–2015.

**Figure 1:**
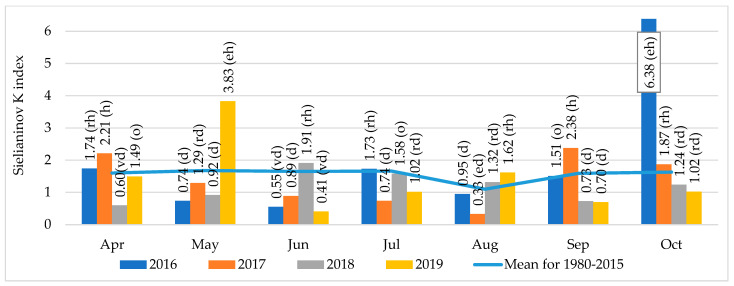
Sielianinov’s hydro-thermic coefficients K described for Poland by Skowera et al. [39]: K ≤ 0.4 extremely dry (ed), 0.4 < K ≤ 0.7 very dry (vd), 0.7 < K ≤ 1.0 dry (d), 1.0 < K ≤ 1.3 relatively dry (rd), 1.3 < K ≤ 1.6 optimal (o), 1.6 < K ≤ 2.0 relatively humid (rh), 2.0 < K ≤ 2.5 humid (h), 2.5 < K ≤ 3.0 very humid (vh), K > 3.0 extremely humid (eh).

Thermal and rainfall conditions during the growing season of the plants were evaluated based on Sielianinov’s hydro-thermal coefficient K (Figure 1). According to this criterion, the vegetation of winter wheat experienced water scarcity conditions from May 2017 until harvest (relatively dry—May, dry—June and July, extremely dry—August). Deficiencies in spring precipitation also occurred in 2018 (very dry—April and dry—May), while hydro-thermal conditions were favourable for wheat in the subsequent period. For this reason, the course of hydro-thermal conditions in 2018 was considered the most favourable for wheat during the entire research period. In contrast, in 2019, thermal and rainfall conditions in April were optimal, and May was extremely humid, after which moisture conditions deteriorated later in the growing season (extremely dry—June, rather dry—July).

### 2.4. Analysis of the Chemical Composition of the Grain

Unground wheat grain was used to assess chemical composition using Raman spectroscopy. The Raman spectra were taken with a Nicolet NXR 9650 FT-Raman spectrometer (Thermo Fisher Scientific, Waltham, MA, USA) equipped with an Nd laser: YAG (1064 nm) and germanium detector. Measurements were carried out in the range from 150 to 3700 cm^−1^ at a laser power of 1 W. An out-of-focus laser beam with a diameter of about 100 μm and a spectral resolution of 8 cm^−1^ was used. Each spectrum was collected using 128 scans. The Raman spectra were analyzed using OPUS 7.0.129 software.

### 2.5. Statistical Analysis

To determine the regularity between the variables (to verify that the changes in chemical composition occurring between the study groups, as measured by Raman spectroscopy, are distinguishable), principal component analysis (PCA) was performed. Obtained Raman spectra give a large amount of data. Therefore, it is necessary to show the most important data. Consequently, PCA was chosen because it is a method for analyzing large datasets with a high number of features per observation. PCA algorithm can increase the interpretability of data, while preserving the maximum amount of information and enabling the visualization of multidimensional data. To summarize, PCA is a statistical technique for reducing the dimensionality of a dataset. In the presented study, we used so-called “fingerprint” region, i.e., between 800 cm^−1^ and 1800 cm^−1^, for PCA analysis. Consequently, we used 259 data from each obtained spectrum. Furthermore, to show which groups and samples were similar to each other, hierarchical cluster analysis (HCA) was performed using the same data as for PCA analysis. HCA analysis was conducted using paired group (UPGMA) algorithm with Euclidean similarity index. Both analyses were carried out using Past 3.0 software.

## 3. Results and Discussion

The present study investigated the possibility of using the Raman spectroscopy technique to evaluate the effect of the foliar application of crop protection products on winter wheat grain quality. Among the measures used, in addition to standard fungicide protection, biostimulants that are increasingly common in modern agriculture were used. In particular, their role is to maximize yields and improve quality, especially under unfavourable environmental conditions for plant growth and development [16,21].

Conventional methods for identifying the chemical composition of cereal grains are time-consuming and labour-intensive, making it difficult to analyze large quantities of samples quickly and economically [40]. The Raman spectroscopy technique provides valuable information on grain chemical composition (including the presence of starch, lipids, protein, cellulose and lignin), as indicated by a study by Yang, et al. [41] performed on corn grain. A measurement using the Raman spectroscopy technique was also performed to identify ash and protein in flour obtained from wheat grain in a study by Czaja, et al. [42].

**Figure 2:**
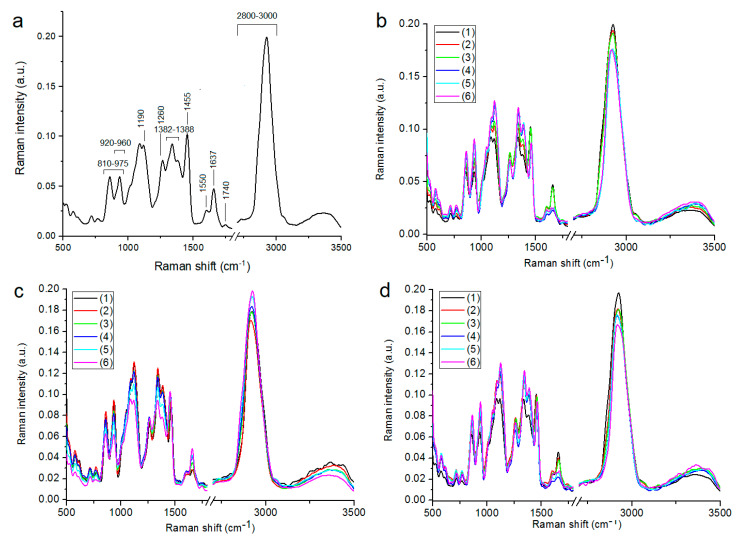
FT-Raman spectra with marked analyzed region (**a**) for wheat grown in 2017 (**b**), 2018 (**c**), and 2019 (**d**); crop protection variants: (1) control (black line); (2) intensive (red line); (3) extensive (green line); (4) PlanTonic BIO (blue line); (5) PlanTonic BIO + Natural Crop (azure line); (6) PlanTonic BIO + Biofol Plex (pink line).

In the conducted experiment, the Raman spectra of wheat grains showed areas characteristic of vibrations of functional groups that build nucleic acids, sugars, proteins and fats. The characteristic Raman intensities of the measured grains are (Figure 2a):

- 810–975 cm^−1^ (stretching vibration of C-C group);
- 920–960 cm^−1^ (vibration of C-C group of amylose);
- 1190 cm^−1^ (vibration of C-C group of sugars);
- 1260 cm^−1^ (vibration of III-row amide—protein);
- 1382–1338 cm^−1^ vibration of the secondary structure of proteins (α-helix);
- 1455 cm^−1^ (vibration of C-H group of proteins and sugars);
- 1550 cm^−1^ (vibration of protein groups included in amylopectins)
- 1637 cm^−1^ (vibration of protein groups included in amylopectins);
- 1740 cm^−1^ (vibration of C-O group of proteins and fats);
- 2800–3000 cm^−1^ (stretching vibration of C-H groups included in carbon chains of fatty acid residues) [42,43].

Compared to the control (wheat grain from facilities without antifungal plant protection) (Figure 2b,d), for 2017 and 2019, grain from the crop with plant protection showed a higher content of C-C group stretching vibrations (810–975 cm^−1^), C-C group vibrations from amylose (920–960 cm^−1^), C-C group vibrations from sugars (1190 cm^−1^), protein-specific amide III vibrations (1260 cm^−1^), protein secondary structure (α-helix) vibrations (1382, 1338 cm^−1^), C-H group vibrations from proteins and sugars (1455 cm^−1^), and a lower content of vibrations of protein groups included in amylopectins (1550 cm^−1^, 1637 cm^−1^), vibrations of C-O group of proteins and fats (1740 cm^−1^), stretching vibrations of C-H groups included in carbon chains of fatty acid residues (2800–3000 cm^−1^). A larger decrease in fat functional groups is observed for 2019. Summarizing the Raman spectroscopic measurements of 2017 and 2019, the application of selected plant protection to wheat resulted in an increase in amylose (included in starch) and other sugars and proteins, and a decrease in amylopectin (included in starch) and fat. With regard to the controls, the grains of winter wheat grown in 2018 (Figure 2c), variants 3, 4, 5 and 6, were characterized by lower contents of C-C group stretching vibrations (810–975 cm^−1^), C-C group vibrations from amylose (920–960 cm^−1^), C-C group vibrations from sugars (1190 cm^−1^), vibrations of the third-row amide—protein (1260 cm^−1^), vibrations of the secondary structure of proteins (α-helix) (1382, 1338 cm^−1^), vibrations of the C-H group from proteins as well as sugars (1455 cm^−1^); a higher content of vibrations of protein groups included in amylopectins (1550 cm^−1^, 1637 cm^−1^), vibrations of the C-O group building proteins as well as fats (1740 cm^−1^). Furthermore, variant 2 showed very similar contents of chemical compounds whose functional groups can be attributed to Raman shift values up to 1740 cm^−1^. In the case of the stretching vibrations of C-H groups included in the carbon chains of fatty acid residues (2800–3000 cm^−1^), it can be seen that intensive protection causes a decrease in their content. In variants 4, 5 and 6, an increase in the content of a stretching C-H groups included in the carbon chains of fatty acid residues (2800–3000 cm^−1^) was observed, while variant 3 did not change the amount of these functional groups in comparison with control one. Summarizing the Raman measurements for 2018, the use of synthetic preparations in variant 2 resulted in a decrease in fatty acids, while in variant 3, it led to an increase in amylopectins, proteins and fat and a decrease in sugars and some proteins.

On average, the analysis of the chemical composition in our study showed an increase in amylose, sugars and proteins and a decrease in fatty acids as a result of the application of crop protection products. A similar relationship was also obtained by Ciolek et al. [44] in a study that compared the impact of growing crops under organic and conventional systems. The grain from cultivation in an organic system, where chemical protection was not used, had a higher content of fatty acids, especially valuable unsaturated acids. The protein content of the wheat grain is an important determinant of its quality, determining the nutritional and rheological properties of dough, which is of great importance in the face of a changing climate. Variable weather conditions prevailing during the experiment may have significantly affected the formation of quality indicators in wheat grain [45,46]. Under the influence of rainfall deficiency and increased temperature, the protein content of the grain increases, as also shown by Hotea et al. [47]. In our study, such a relationship was observed in 2019, when there was a shortage of precipitation in the grain filling phase. The most favourable conditions for starch accumulation in wheat grain prevailed in 2019. Similar to Cociu and Alionte [48], it has been shown that greater starch accumulation is particularly favoured by warm and dry conditions during grain maturation.

**Figure 3:**
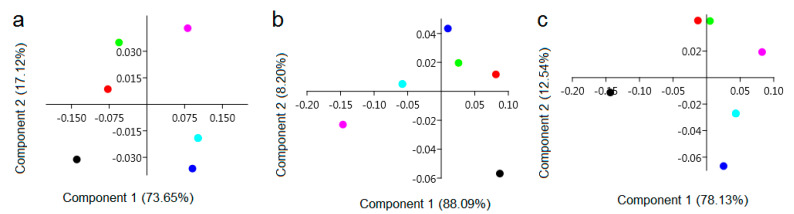
PCA analysis of FT-Raman spectra of wheat grains grown in 2017 (**a**), 2018 (**b**), 2019 (**c**); crop protection variants: (1) control (black dot); (2) intensive (red dot); (3) extensive (green dot); (4) PlanTonic BIO (blue dot); (5) PlanTonic BIO + Natural Crop (azure dot); (6) PlanTonic BIO + Biofol Plex (pink dot).

The paper also presents the results of a principal component analysis (PCA), the preferred method for extracting features that is used to reduce the size of a set of features [49], and a hierarchical cluster analysis (HCA) is used to group them [50]. PCA analysis shows that each of the crop protection options significantly affects the chemical composition of winter wheat grain. This is a trend observed in each year of the study. In addition, in 2017, a similar effect on chemical composition was found in variants 2 and 3, with synthetic fungicides and biostimulant application in variants 4 and 5 (Figure 3a). In 2018, protection variants 2 and 3 and the biostimulant in variant 4 had similar effects on the chemical composition of wheat grain (Figure 3b). In 2019, similarity in chemical composition was observed for wheat grain treated with biostimulants in variants 4, 5 and 6, as well as in variant 3 using synthetic formulations (Figure 3c).

**Figure 4:**
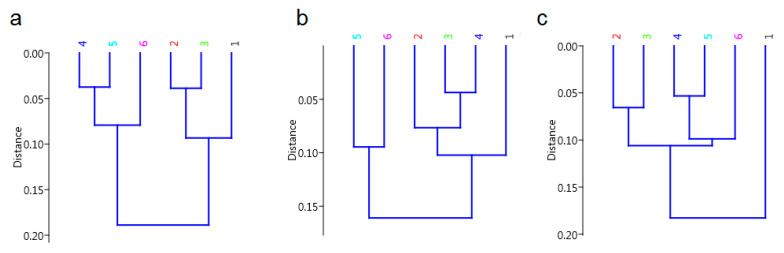
HCA analysis of FT-Raman spectra of wheat grains grown in 2017 (**a**), 2018 (**b**), 2019 (**c**); crop protection variants: control (1); intensive (2); extensive (3); Plantonic (4); Plantomic + Natural Crop (5); Plantonic + Biofol Plex (6).

The results of the HCA analysis for each of the years analyzed indicate the formation of two groups of similar plants (in terms of chemical composition). In addition, each year, the separation of grain from plants without the application of preparations is observed, which means that its chemical composition significantly differs from that observed for grains in facilities with preparations for protection against fungal diseases. In 2017, wheat grains from variants 2 and 3, in which plants were protected with synthetic preparations, were found to be similar in chemical composition. In addition, plants treated with a Plantonic biostimulant in each configuration (variants 4, 5 and 6) show similar grain chemistry (Figure 4a). In 2018, one can see a similarity in the chemical composition of grain after the application of synthetic preparations in variants 2 and 3, and in variants 4, 5 and 6, in which biostimulants were used for plant protection (Figure 4b). In 2019, the first group of similarities is grains from variants 2 and 3, while the second group of similarities is grains after biostimulant application (variants 4, 5 and 6) (trend as in 2017) (Figure 4c).

## 4. Conclusions

The study confirms the applicability of the Raman technique characterized by fast and non-destructive measurements to assess the quality of winter wheat grain. Using this measurement technique, the effect of applied crop protection products, including biostimulants, and hydro-thermal conditions during the years of the study on the chemical composition of wheat grain was demonstrated. It was indicated that hydro-thermal conditions and applied biostimulants changed the chemical composition of the grain during the studied years. The results of the HCA analysis for each of the years analyzed indicate the formation of two groups of similar plants in terms of their chemical composition. Wheat grains of plants treated with synthetic preparations in variants with intensive and extensive protection were found to be similar in chemical composition. The second group of similarities in terms of chemical composition are the grains of plants treated with biostimulants (PlanTonic BIO, PlanTonic BIO + Natural Crop and PlanTonic BIO + Biofol Plex), which caused an increase in fatty acid content.

The future of using biostimulants in crop production, including those containing SA and nettle extracts, appears to be a promising alternative, despite their often weaker action against phytopathogenic organisms compared to synthetic crop protection products. This research could inspire further studies on the effect of biostimulants on the quality of seed yield in other crop species.

## Figures and Tables

**Table 1:** Conventional plant protection products and biostimulants used in the cultivation of winter wheat—doses and dates of application.

Plant Protection	Treatment Time (BBCH Scale)	Trade Name	Active Substance (gdm^−3^)	Terms and Doses	
				Preparation (dm^3^ha^−1^)	Active Substance (gha^−1^)
(1) Control (without plant protection)	-	-	-	-	-
(2) Intensive	31–33	Duett Star 334 SE (BASF)	Fenpropimorph (250)	1.0	250
			Epoxiconazole (84)		84.0
	45–47	Acanto 250 SC (DuPont)	Azoxystrobin (250)	0.6	150
		Bumper 250 SC (ADAMA)	Propiconazole (250)	0.7	175
	65–69	Mystic 250 EC (Nufarm)	Tebuconazole (250)	0.9	225
(3) Extensive	31–33	Duett Star 334 SE (BASF)	Fenpropimorph (250)	1.0	250
			Epoxiconazole (84)		84.0
	45–47	Acanto 250 SC (DuPont)	Azoxystrobin (250)	0.6	150
		Bumper 250 SC (ADAMA)	Propiconazole (250)	0.7	175
(4) PlanTonic BIO	31–33	PlanTonic BIO	No data	4.0	No data
	45–47			4.0	
	65–69			4.0	
(5) PlanTonic BIO + Natural Crop	31–33	PlanTonic BIO		4.0	
		Natural Crop SL		1.5	
	45–47	PlanTonic BIO		4.0	
		Natural Crop SL		1.5	
	65–69	PlanTonic BIO		4.0	
		Natural Crop SL		1.5	
(6) PlanTonic BIO + BioFol Plex	31–33	PlanTonic BIO		4.0	
		BioFol Plex		2.0	
	45–47	PlanTonic BIO		4.0	
		BioFol Plex		2.0	
	65–69	PlanTonic BIO		4.0	

**Table 2:** Chemical composition of biostimulants used in the experiment.

Biostimulators	Biostimulator Charakteristics	
BioFol Plex (Biostyma, Września, Poland)	Complexed with humic acids	2.0% N_tot_; 0.3% Mg; 5.0% S; 0.15% B; 0.05% Cu; 0.20% Fe; 0.10% Mn; 0.50% Zn; 1.25% C; 5.0% extract from algae; traces of plant hormones, betaine (C_5_H_11_NO_2_), plant-derived amino acids, thiamine
Natural Crop SL (Natural Crop, Italy)	Concentrate of peptides and L-amino acids obtained by enzymatic hydrolysis of collagen	9.0% NO_2_^−^-N; 24.5% C_org_; total > 50%, free > 2.0% L-amino acids (GLY, PRO, HYP, GLU, ALA, ARG, ASP, SER, HIS, LYS, LEU, VAL, PHE, ILE, THR, TYR, CYS, MET)
PlanTonic Bio (OGET Innovations GmbH, Allerheiligen bei Wildon, Austria)	Nettle (*Urtica dioica* L.) extract, willow (*Salix* sp.) extract, sunflower (*Helianthus annus* L.) oil	Full chemical composition of the preparation reserved by the manufacturer

**Table 3:** Doses and dates of application of fertilisers.

Type of Fertiliser	Trade Name	Chemical Composition	Fertiliser Dose (kg ha^−1^)	Dose (ha^−1^ Year^−1^)	Application Term
Soil-applied	Polifoska 6	6% N-NH_4_^+^, 20% P_2_O_5_, 30% K_2_O, 7% SO_3_	350	21 kg N, 70 kg P_2_O_5_, 105 kg K_2_O, 24.5 kg SO_3_	before sowing
	Saletrosan^®^ 26	26% N (including 19% N-NH_4_^+^, 7% N-NO_3_^−^), 32.5% SO_3_	300	88.4 kg N, 110.5 kg SO_3_	BBCH 25–27
	Zaksan^®^	32% N (including 16% N-NH_4_^+^, 16% N-NO_3_^−^),	250	80 kg N	BBCH 33–35
Foliar fertilisers	GranuFol Fosfor	10.0% N, 41% P_2_O_5_, 12% K_2_O, 2.3% MgO, 2.5% SO_3_, 0.03% B, 0.03% Cu, 0.16% Fe, 0.07% Mn, 0.002% Mo, 0.07% Zn	1.0	10 g N, 410 g P_2_O_5_, 120 g K_2_O, 23 g MgO, 25 g SO_3_, 0.3 g B, 0.3 g Cu, 1.6 g Fe, 0.7 g Mn, 0.02 g Mo, 0.7 g Zn	BBCH 31–32
	GranuFol CuMan	43.3% SO_3_, 5% Cu, 25% Mn	0.3	129.9 g SO_3_, 15 g Cu, 75 g Mn	
	Wuxal mikro	5% N, 10% K_2_O, 3% MgO, 5.2% S, 0.3% B, 0.5% Cu, 1% Fe, 1.5% Mn, 0.01% Mo, 1% Zn	0.5	39 g N, 78.5 g K_2_O, 23.5 g MgO, 40.5 g S, 2.35 g B, 3.95 g Cu, 7.85 g Fe, 11.8 g Mn, 0.75 g Mo, 7.85 g Zn	
	Granufol Mag	20% MgO, 41% SO_3_, 0.42% Fe	1.5	600 g MgO, 1230 g SO_3_, 12.6 g Fe	BBCH 31–32 BBCH 54–55
	GranuFol Potas	10.0% N, 12% P_2_O_5_, 41% K_2_O, 2.3% MgO, 2.5% SO_3_, 0.03% B, 0.03% Cu, 0.16% Fe, 0.07% Mn, 0.002% Mo, 0.07% Zn	1.0	10 g N, 120 g P_2_O_5_, 410 g K_2_O, 23 g MgO, 25 g SO_3_, 0.3 g B, 0.3 g Cu, 1.6 g Fe, 0.7 g Mn, 0.02 g Mo, 0.7 g Zn	BBCH 54–55

## Data Availability

Not applicable.
